# High-Throughput Automated Microscopy of Circulating Tumor Cells

**DOI:** 10.1038/s41598-019-50241-w

**Published:** 2019-09-24

**Authors:** Carlos Aguilar-Avelar, Brenda Soto-García, Diana Aráiz-Hernández, Juan F. Yee-de León, Miguel Esparza, Franco Chacón, Jesús Rolando Delgado-Balderas, Mario M. Alvarez, Grissel Trujillo-de Santiago, Lauro S. Gómez-Guerra, Liza P. Velarde-Calvillo, Alejandro Abarca-Blanco, J. D. Wong-Campos

**Affiliations:** 1 Delee Corp., Mountain View, CA 94041 USA; 2 Departamento de Bioquímica y Medicina Molecular, Facultad de Medicina, Universidad Autónoma de Nuevo León, Monterrey, 64460 Mexico; 3 Centro de Biotecnología-FEMSA, Escuela de Ingeniería y Ciencias, Tecnologico de Monterrey, Monterrey, 64849 Mexico; 4 Departamento de Bioingeniería, Escuela de Ingeniería y Ciencias, Tecnologico de Monterrey, Monterrey, 64849 Mexico; 5 Departamento de Mecatrónica e Ingeniería Eléctrica, Escuela de Ingeniería y Ciencias, Tecnologico de Monterrey, Monterrey, 64849 Mexico; 6 Servicio de Urología, Hospital Universitario “Dr. José Eleuterio González”, Universidad Autónoma de Nuevo León, Monterrey, 64460 Mexico; 7 Department of Physics, Joint Quantum Institute and Joint Center for Quantum Information and Computer Science, University of Maryland, College Park, MD 20742 USA

**Keywords:** Cancer imaging, Translational research, Biomedical engineering

## Abstract

Circulating tumor cells (CTCs) have the potential of becoming the gold standard marker for cancer diagnosis, prognosis and monitoring. However, current methods for its isolation and characterization suffer from equipment variability and human operator error that hinder its widespread use. Here we report the design and construction of a fully automated high-throughput fluorescence microscope that enables the imaging and classification of cancer cells that were labeled by immunostaining procedures. An excellent agreement between our machine vision-based approach and a state-of-the-art microscopy equipment was achieved. Our integral approach provides a path for operator-free and robust analysis of cancer cells as a standard clinical practice.

## Introduction

Circulating tumor cells (CTCs) are cancer cells that have been shed from a primary or metastatic solid tumor and are carried around the bloodstream of cancer patients. They play a fundamental role in the metastatic process of non-hematological cancers^1–3^ and hold the potential of becoming a blood-based biomarker that can predict, diagnose and guide clinical decisions^4–11^. Moreover, phenotypic and genotypic analysis of CTCs can enable the continuous assessment of mutations and allow treatment personalization^12–17^. Unfortunately, most of the current technologies for CTC capture leave patient samples with a high background of contaminating cells, since a few CTCs (∼10) need to be found on a background of 10^10^ cells^18–20^. Furthermore, high heterogeneity between CTCs exacerbate the difficulty of its capture and characterization^21,22^.

Typical methods for CTCs identification and analysis make use of fluorescence microscopy and immunofluorescence techniques, by imaging specific markers that depend on the phenotypes of the tumor cells^23–32^. The complexity of such approaches is that it requires manual counting and analysis by trained technicians that are prone to develop biased criteria and fatigue over time, which can corrupt or mislead conclusions based on data. To achieve reliable reproducibility and deterministic interpretation it is required the implementation of a framework that can handle high data throughput in both, the hardware and software, while minimizing human intervention^33^.

This article describes the construction and validation of a fully automated microscope for its application in the recognition of CTCs in a blood sample. The microscope hardware was designed to accurately discriminate CTCs among cells present in a blood sample. The hardware was optimized for the proper fluorophore excitation and efficient capture of light emitted from stained cells. Finally, fluorescence signals were used to automatically classify and precisely identify the positions of few CTCs on a sample with thousands of background cells distributed on a 10 by 10 mm area.

We show two sets of experiments. First, the microscope counting and autofocusing capabilities were validated using standard methods, against state-of-the-art microscopy equipment. The second experiment demonstrates a realistic application of our microscope to the imaging of prostate cancer cells stained with four different markers. The most important characteristic of our imaging system is that it can be incorporated directly into the sample analysis workflow. Furthermore, its hardware and software are optimized for the intended application, resulting in a cost-effective, high-performance and highly-customizable solution for the automatic imaging, classification and counting of CTCs. Therefore, the main advantage of our microscope lies on its high data throughput, image recognition capabilities, and a user-free approach immune to operator error, which clears the path for CTC analysis as a standard clinical practice.

## Material and Methods

### Microscope hardware

#### Optics

**Figure 1:**
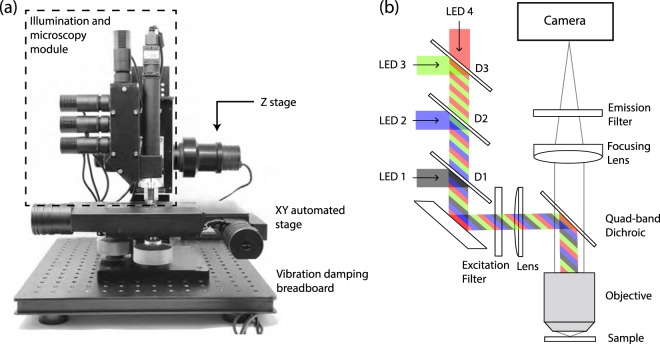
(**a**) Experimental setup. The illumination and microscopy modules are attached to a Z stage on a XY translation stage. Vibration damping is provided by four Sorbothane feet. (**b**) Diagram of the illumination and microscopy module. The microscope consist of four commercial LEDs (LED 1 through 4) epicospically illuminating the microscope objective at 375, 470, 565, 625 nm, respectively. Fluorescence from the cells is collected with the same microscope objective and imaged by a CMOS camera.

We designed and built a four color microscope as described in Fig. 1. Our microscope illumination system consists of four commercial LEDs at peak wavelengths of 375 nm (Thorlabs, M375L3), 470 nm (Thorlabs, M470L3), 565 nm (Thorlabs, M565L3), and 625 nm (Thorlabs, M625L3). Light is collimated with an aspheric lens (Thorlabs, ACL2520U) and sent to the reflective side of a quad-band dichroic filter (Semrock, FF409/493/573/652-Di02-25x36) facing the sample through an infinity corrected microscope objective (Parco Scientific, 58A-0645 20X Infinity Plan Achromatic). The selectivity at each excitation wavelength is provided by three long-pass dichroic mirrors D1 (Thorlabs, DMLP425R), D2 (Thorlabs, DMLP490R), and D3 (Thorlabs, DMLP605R) with cut-off wavelengths of 425 nm, 490 nm, and 605 nm, respectively, which separate the excitation light in four different bands. The excitation light is focused to the backside of the microscope objective with a plano-convex lens (Thorlabs, LA1255-A), producing an even illumination at the frontal focal plane, following the Köhler illumination technique. The fluorescence emitted from the cells upon excitation is transmitted through the objective and imaged by an achromatic lens on a CMOS camera. Emission and excitation filters were placed on the opposite sides of the dichroic, since it was experimentally found that it improves the signal to noise ratio of the image. The optical setup described above was mounted on the 3D printed body, as depicted in Supplementary Fig. S6.

#### Camera

A 20 MP CMOS camera (PointGrey, BFS-U3-200S6M-C) was selected due to the combination of adequate noise parameters, sensor characteristics, and its potential to be used in large area widefield microscopy. Moreover, this camera supports sensor pixel binning which increases the camera sensitivity at the expense of resolution. The signal (S) recorded at the sensor for an exposure time of *t* can be calculated from the photon flux (*γ*) reaching the squared area of each pixel with size *δ* and the ratio of signal electrons generated per photon, known as quantum efficiency (QE):

1 $$S(t)=\gamma {\delta }^{2}{\rm{QE}}t\mathrm{.}$$

The signal is fundamentally degraded by the noise (N) that is described as

2 $$N(t,T)=\sqrt{{\sigma }_{{\rm{camera}}}{(t,T)}^{2}+{\sigma }_{{\rm{shot}}}{(t)}^{2}},$$

where ${\sigma }_{{\rm{shot}}}(t)=\sqrt{S(t)}$ is the photon shot noise produced by the random nature of light reaching the sensor area and ${\sigma }_{{\rm{camera}}}(t,T)={\sigma }_{{\rm{dark}}}(T)+{\sigma }_{{\rm{read}}}(t)$ is the added noise of the camera, that includes the dark shot noise due to the camera temperature *T* and the read noise of the camera electronics. It is important to remark that Eq. (2) holds for uncorrelated noise sources.

The chosen camera has an IMX183 sensor with QE of 79% at 525 nm, a total sensor area of 13.13 × 8.76 mm, and pixel size of *δ* = 2.4 *μ*m. The camera read noise has a value of ${\sigma }_{{\rm{camera}}}(t,T)$ = 3.30 *e*^−^ (electrons), which sets the signal level that equates the noise at 4.83 photons.

#### Mechanics

We used a motorized stage (Prior Scientific, ProScan II H101) with a travel range of 114 × 75 mm, 2.2 *μ*m of bi-directional repeatability and a minimum step size of 0.04 *μ*m on the XY axis, and a linear Z stage (Nikon, Focus Block model L-IM) for focus adjustment with 2.2 *μ*m of bi-directional repeatability and a resolution of 0.002 *μ*m. Both stages were driven using a three-axis stage controller (Prior Scientific, ProScan II H30) through serial communication with G code commands. This setup was mounted on an optical breadboard with four Sorbothane feet that provide vibration damping (Fig. 1a).

#### Illumination driver

The necessary electronics to drive the LEDs was custom built. The driver electronics is based on the pulse width modulation power driver CAT4101, which is controlled by an 8-bit microcontroller (Arduino nano), able to provide up to 1 A of current to each LED. Pulse width modulation of the input signal allows different brightness settings with a resolution of 8 bits. The schematic diagram of this driver is given in Supplementary Fig. S7.

The optical power emitted at the microscope objective’s front focal plane is 23.50, 34.51, 10.95 and 41.58 mW for the 375, 470, 565 and 625 nm color bands driven at 0.9 A, respectively. We found that roughly 5% of the output light from each LED coupled to the microscopy objective due to the incoherent nature of the LEDs and lossy optical path. Optical loss is exacerbated for the 565 nm LED due to spectral band-pass filtering of the emission, which halves the input power at the microscope back plane.

### Image processing software

#### Classification algorithm

**Figure 2:**
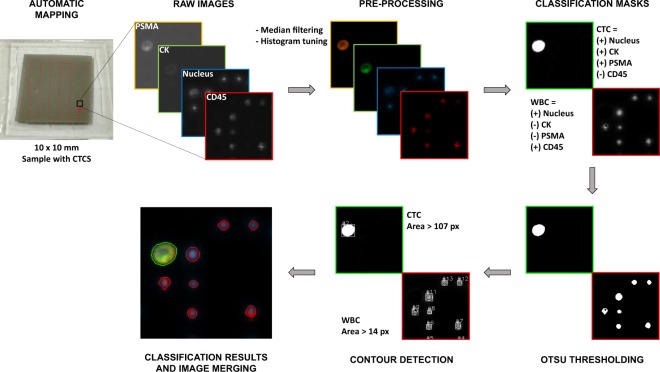
Image processing workflow. A human blood sample was spiked with 100 cancer cells, processed through a filtering membrane, and imaged using our microscope. Four images per field of view are taken, one at each excitation wavelength, and collect the emission to a grayscale camera. Median filtering and histogram tuning for background reduction and contrast enhancement are applied. The CTC and WBC classification masks are computed and the images are thresholded for the sorting and counting algorithm. Processed images are colorized for display purposes.

We implemented an automated cell recognition and counting routine using OpenCV^34^ for C# in Microsoft Visual Studio, which is capable of distinguishing tumor cells from a background of contaminating peripheral blood cells trapped on a filtering membrane. The recognition algorithm is based on Otsu thresholding, which is a threshold algorithm for picture segmentation that automatically selects the optimum value to separate two classes in a gray-level image^35^, and contour detection that process the images for obtaining the cell size and position. Figure 2 depicts the image processing pipeline, implemented as follows: (1) The raw gray-scale images are acquired from the microscope on each color channel, (2) a median filter is applied and a window/level histogram equalization is performed for background reduction and contrast enhancement, (3) the CTC and WBC classification masks are computed by the pixel-to-pixel addition/subtraction of the different color channels, according to its biological definition; (4) Otsu thresholding is applied to the classification masks and (5) the contours in the thresholded images are computed and discriminated by size. (6) Finally, a merged and colored image is generated for visualization of the final cell labels. Therefore, the recognition algorithm for the cell classification is performed according to appearance channel, brightness and size of the cells.

#### Autofocus

We implemented a brightness-based autofocus approach, which relies on the fact that the cells brightness will be maximum when the image is in focus, assuming that the image is not over-exposed. First, a range on the Z-axis is predefined, where the image of maximum brightness will be searched and selected. This range should be as extensive as needed to ensure that the object of interest is within the range. Then, a predefined quantity of equidistant pictures is taken so that the full range is covered, and the maximum brightness value is computed for every picture. Finally, the image with maximum global brightness is selected. A flowchart with a detailed description of the autofocus algorithm and its parameters is given in Supplementary Fig. S1. This approach is used for both, finding the cells location within a wide-range on the Z-axis (coarse adjustment of the focus plane) and correcting small variations due to the sample shape and position (fine adjustment). A performance validation of the autofocus algorithm is shown in the Supplementary Fig. S2, where our brightness-based approach is compared against a contrast-based focus function^36^, demonstrating that our focus function outperforms the contrast-based approach for the imaging of almost empty samples, while the yield of the two focus descriptors is comparable for the case of well-populated samples. Furthermore, Supplementary Fig. S3 depicts the results of the imaging of a tilted sample with and without autofocus.

#### Stitching

The limited field of view of the objective is compensated by an automated mapping routine which makes possible the imaging of larger samples. For the XY-axis stitching of the images, it was considered that the camera captures an area equal to 818 × 547 *μ*m with a resolution 1368 × 912 pixels using a 4 × 4 pixel binning. Considering this relation, and taking advantage of the 2.2 *μ*m bi-directional repeatability of the motorized stage, a simple mechanical alignment was performed and the automatic mapping routine captures contiguous zones, enabling the generation of a panoramic picture of the sample. On the Z axis, the autofocus routine ensures that larger samples are imaged uniformly. The performance of the stitching algorithm is shown in Supplementary Fig. S3.

### Sample preparation

#### Two-color sample preparation

PC-3 cancer cell cultures were stained with 25 uM of CellTracker orange CMRA (Thermo Fisher Scientific, Waltham, Massachusetts, USA) and incubated them for 45 minutes at 37 in an air atmosphere of 5% CO2. Cells were harvested using a trypsin-EDTA solution (Thermo Fisher Scientific, Waltham, Massachusetts, USA) and then counted with a hemocytometer. The stained cell suspension was serially diluted to achieve a concentration of approximately 100 cells per 30 *μ*l, that volume was then added to 7.5 ml of a whole blood sample collected from a healthy donor. The spiked blood sample was diluted with a 0.3% formaldehyde - 0.15% pluronic F68 solution in PBS to a 1:2 v/v ratio and incubated for 10 minutes at room temperature. Afterward, the blood sample was processed by a membrane-based microfiltration device that captures CTCs due to differences in size and deformability with regard blood cells. After processing the blood sample, PBS was flowed to clear residual peripheral blood cells remaining in the membrane. Fixation and nuclear staining were carried out by passing 1 ml of 4% formaldehyde and 1 ug/ml Hoechst 33342 solution, respectively, followed by an incubation of 10 minutes and a washing step after the introduction of each reagent. Finally, the membrane was mounted on a microscope slide using Fluoromount-G (Thermo Fisher Scientific, Waltham, Massachusetts, USA) for its subsequent analysis by fluorescence microscopy.

#### Four-color sample preparation

A blood sample of 7.5 ml from a healthy donor was spiked with 100 LNCaP cancer cells. The spiked blood sample was diluted with a 0.3% formaldehyde - 0.15% pluronic F68 solution in PBS to a 1:2 v/v ratio and incubated for 10 minutes at room temperature. Afterward, the blood sample was processed through the microfiltration device and PBS was flowed to clear residual peripheral blood cells remaining on the filtering membrane. Fixation was performed by flowing 1 ml of 4% formaldehyde in PBS followed by a 10-minute incubation. Subsequently, permeabilization was carried out by passing 1 ml of 0.3% PBST solution followed by a 10-minute incubation. Afterward, to prevent non-specific binding of antibodies, blocking was made by flowing 1 ml of 1% BSA in PBS followed by a 30-minute incubation. Then, 500 ml of an antibody cocktail containing 8 mg/ml of Alexa Fluor 488 labeled anti-cytokeratin (pan reactive) (clone C-11), Alexa Fluor 647 labeled anti-human CD45 (clone HI30) and biotin labeled anti-human PSMA (FOLH1) (clone LNI-17) antibodies (BioLegend, San Diego, California, USA) in 0.1% PBST solution was passed, followed by an hour incubation. Afterward, 500 ml of a mixture containing 5 mg/ml of Hoechst 33342 and 8 mg/ml of streptavidin-Alexa Fluor 647 conjugate (Thermo Fisher Scientific, Waltham, Massachusetts, USA) in 0.1% PBST solution was flowed, followed by 1 hour of incubation. After finishing each incubation with the solutions described above, washing steps using PBS were conducted at 500 ul/min for 5 minutes. Finally, the membrane was mounted on a microscope slide using Fluoromount-G (Thermo Fisher Scientific, Waltham, Massachusetts, USA).

#### Human participants and ethical approval

Blood samples from healthy donors were collected in BD Vacutainer K2EDTA blood collection tubes (BD, Franklin Lakes, New Jersey, USA). Informed consent was acquired from the donors and ethical approval was granted by the Hospital Universitario “Dr. José Eleuterio González” - UANL, Mexico (Research Ethics Committee Approval no. UR16-0007). All methods were performed in accordance with the relevant ethical guidelines and regulations.

## Results and Discussion

### Imaging and counting of pre-stained cancer cells

**Figure 3:**
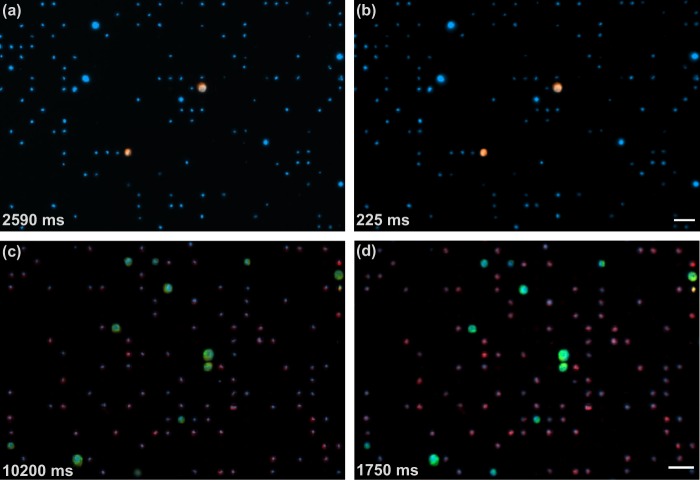
Visual comparison between the images of our automated microscope and the Axio Observer. Top sub-figures are two-color snaps of the sample showing pre-stained PC-3 cancer cells emitting blue and orange light, and nucleated blood cells emitting only blue light, which were imaged with (**a**) the Axio Observer and (**b**) our automated microscope. Bottom sub-figures are four-color snaps of the sample with immunostained LNCaP cancer cells and blood cells which were imaged with (**c**) the Axio Observer and (**d**) our microscope. Bottom-left numbers show the total exposure time of each picture. Scale bars: 50 *μ*m.

In this experiment, our device was characterized by imaging pre-stained tumor cells which were introduced into a blood sample that was later processed through a microfiltration device. In particular, a sample containing prostate cancer cells (PC-3) that were pre-stained with CellTracker and nuclei stained with Hoechst 33342 was imaged. The sample was prepared as described in the Materials and Methods section. Two-color snaps (CellTracker in orange and Hoechst 33342 in blue) were captured and the cells were counted from 15 different regions of the sample. The blue images were captured with 25 ms of exposure time and the orange images were captured with 200 ms of exposure time. By imaging the same 15 regions of the membrane, the performance of our setup was compared with the Axio Observer (ZEISS, Oberkochen, Germany), equipped with a motorized stage, a Colibri 7 LED light source (wavelengths 385, 475, 555, 630), an Axiocam 506 mono, 90 HE DAPI/GFP/Cy3/Cy5 filter set, and a Plan-NEOFLUAR 10X/0.3 objective. For the Axio Observer, the blue images were captured with 90 ms of exposure time and the orange images were captured with 2500 ms of exposure time. A visual comparison between the images captured with (a) the Axio Observer and (b) our microscope is shown in Fig. 3. As can be seen, both images can be used for the manually counting of CTCs and nuclei. However, it is remarkable that the total exposure time of the two-color images captured with our microscope was an order of magnitude smaller than the exposure of the images acquired with the Axio Observer.

**Figure 4:**
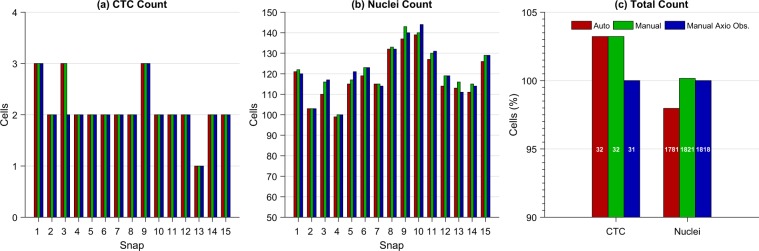
Results of the automatic and manual classification and counting obtained from the two-color images acquired using the automated microscope and the Axio Observer. (**a**) Results of the CTC count per snap. (**b**) Results of the nucleated cells count per snap. (**c**) Results of the total CTC and nucleated cells counts expressed in percentage with respect to the manual count performed from the Axio Observer images. The results of the automatic and manual counts made from the images taken with our microscope are labeled as ‘Auto’ and ‘Manual’, respectively, and the results of the manual count performed from the images taken with the Axio Observer are labeled as ‘Manual Axio Obs.’. The white numbers inside the bars in (**c**) indicate the actual cell count.

The parameters for the automatic classification and counting for this experiment were as follows: CTCs are considered as cells imaged in the orange channel, with a nucleus visible in the blue channel, and with an area greater than 107 pixels, which is the area of a circle of 7 *μ*m of diameter. The rest of the cells imaged in the blue channel with an area greater than 14 pixels, which is the area of a circle of 2.5 *μ*m of diameter, are considered as nucleated cells from peripheral blood. As comparison, blind visual classification and manual counting were also performed by a trained technician using FIJI^37^ from both the images acquired using our microscope and the Axio Observer. The results are presented in Fig. 4.

It is worth mentioning from the results of the comparison shown in Fig. 4 that the percentage of CTCs that were automatically and manually counted from our microscope images with respect to the manual count performed from the state-of-the-art microscope images was 103.22%, which represents a difference of only one CTC in the total count. That difference is due to the orange channel in Snap 3 was actually better imaged by the automated microscope than by the Axio Observer, allowing the finding of one additional CTC. On the other hand, the automatic count of nucleus with respect to the manual count was underestimated by 2%. The main reason of that small difference in the count is that some cells are too close to be counted as separated contours. However, this can be corrected by including a more complex algorithm for contour recognition or by training a machine learning model. Finally, it is important to mention that the comparison between the two manual counts of nucleus was 100.165%, representing a difference of only 3 cells of the total count, but the sum of the absolute error per snap was 23 cells, representing a 1.26% with respect to the total count from the Axio Observer images. That difference may be due to human error in the counting and actual differences in the images, as brightness and focus of the cells.

### Imaging and counting of four-color immunostained samples

In addition, a second experiment was carried out for evaluating the performance of our microscope in an application closer to the imaging of CTCs from real patient samples. In this case, a human blood sample spiked with prostate cancer cells (LNCaP) was processed using an immunostaining procedure with four fluorophores (described in the Materials and Methods section), which are used to distinguish the biological properties of tumor cells with respect to blood cells. Namely, cancer cells and blood cells were stained using Hoechst 33342 for the nucleus (blue), Alexa Fluor 488 for cytokeratin (green), Alexa Fluor 568 for PSMA (orange) and Alexa Fluor 647 for CD45 (red), with emission peaks described at Supplementary Fig. S4. Additionally, Supplementary Fig. S5 shows the characterization of the bleed-through of the four color channels with the fluorophores mentioned above, where it is shown that the maximum crosstalk between channels is about 11.97% with respect to the main intensity of channel. All the rest of the values were zero or smaller than 0.5%.

We considered a CTC as a nucleated cell that express epithelial proteins such as cytokeratins 8, 18 and/or 19, and the prostate specific membrane antigen (PSMA), while being negative for the leukocyte-specific antigen CD45^38,39^. Therefore, cancer cells are expected to express blue, green, and orange labels; WBCs should express blue and red colors, while other blood cell populations should be imaged only in blue color. This immunostaining procedure is compatible to be applied to the staining of CTCs from a patient blood sample. Therefore, this test serves as an approach of our technology to the classification and counting of real CTCs.

**Figure 5:**
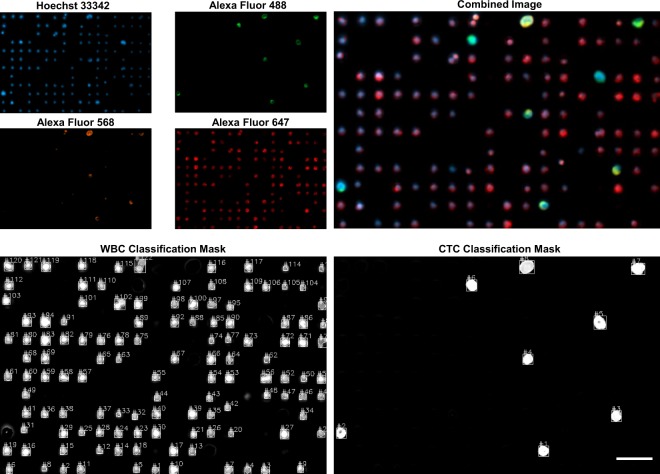
Four-color image acquisition with counting results. Cells were stained with Hoechst 33342 for the nucleus, Alexa Fluor 488 for cytokeratin, Alexa Fluor 568 for PSMA and Alexa Fluor 647 for CD45. The images are analysed looking for tumor cells and combined for visualization. Scale bar of 50 *μ*m.

Four-color snaps were captured from 10 different regions of the sample. The images were acquired using exposure times of 50 ms for the blue, 100 ms for the red, 100 ms for the green, and 1500 ms for the orange. Again, the performance of our automated setup was compared against the Axio Observer microscope described before. The images acquired with this microscope were captured using exposure times of 200 ms for the blue, 3000 ms for the red, 2500 ms for the green, and 4500 ms for the orange. Figure 3 shows a visual comparison of the images acquired with our microscope (d) and the Axio Observer (c). It is worth noting that total exposure time of the images captured with our microscope in four colors was six times smaller than the exposure of the images acquired with the Axio Observer. Additionally, a sample snap is shown in Fig. 5, where each color image can be seen individually and the final picture after the color merging can be also observed. As depicted, manual classification and counting can be easily performed by a trained technician.

**Figure 6:**
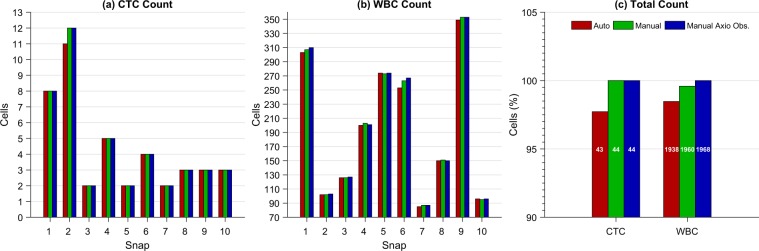
Results of the automatic and manual classification and counting obtained from the four-color images acquired using the automated microscope and the Axio Observer. (**a**) Results of the CTC count per snap. (**b**) Results of the WBC count per snap. (**c**) Results of the total CTC and WBC counts expressed in percentage with respect to the manual count performed from the Axio Observer images. The results of the automatic and manual counts made from the images taken with our microscope are labeled as ‘Auto’ and ‘Manual’, respectively, and the results of the manual count performed from the images acquired with the Axio Observer are labeled as ‘Manual Axio Obs’. The white numbers inside the bars in (**c**) indicate the actual cell count.

The parameters for the automatic classification and counting for this experiment were the following: CTCs are considered as cells with emission in the blue, green and orange channels, and with a size of at least 7 *μ*m of diameter. WBCs are considered as cells with a expression of the blue and red channels, with a minimum size of 2.5 *μ*m. In Fig. 5, the CTC and WBC classification masks generated by our cell recognition algorithm are shown. Our approach allows good discrimination between the two cell classes. As comparison, visual classification and manual counting were also performed by a trained technician using FIJI^37^ from both the images acquired using the our microscope and the Axio Observer. The results of the automatic count and its comparison with the manual counts are presented in Fig. 6.

Figure 6 shows the automatic count of CTCs with respect to the manual counts performed 97.72% of cell recognition, which represents a difference of only one CTC in the total count. That difference is due to Snap 2, where there are two CTCs that are too close to be counted as separated contours. On the other hand, the error of the automatic count of WBCs was −1.52% with respect to the manual count performed from the images captured by the Axio Observer. Finally, it is important to mention that the comparison between the two manual counts showed zero error in the CTCs count and −0.40% in the WBCs count, which represents a difference of only 8 cells of the total count. The sum of the absolute error per snap was 14 cells, representing a underestimation of −0.71% with respect to the total count from the Axio Observer images. Despite the slight underestimation error of the cell recognition algorithm, its simplicity yields a high throughput during the analysis at low computing requirements.

### Throughput assessment

A complete mapping and characterization of the membrane taken with our automated microscope is performed in approximately 30 minutes with the autofocus disabled and 1.5 hours when enabled (15 sample pictures per snap). On the other hand, the images of the Axio Observer microscope, which has no autofocus capabilities and must be operated by a trained technician, are taken in about 4 hours. This is because some regions of the membrane are out of focus and they must be manually re-focused and captured in several independent sub-mappings.

Furthermore, a trained technician requires between 10 and 12 hours to manually enumerate the cells on a single membrane, while our automated microscope return the classification and counting results as soon as the mapping is finished. Therefore, our automated approach for the imaging and classification of CTCs yields a result up to ten times faster than the conventional imaging procedures.

## Conclusions

The automated microscope implemented here demonstrates a pathway for microscopy without human operation with potential applications in cancer diagnosis, prognosis, and treatment personalization. The routines implemented here involved the sampling and refocusing of the specimens with the aid of computer vision and mechanical automation. We found excellent agreement between manual and automated counting on images using our microscope and a state-of-the-art optical microscope. Specifically, classification rates of about 98% and exposure times up to ten times smaller in comparison to state-of-the-art equipment were achieved with the proposed setup. Future work could involve the implementation of machine learning algorithms to make the discrimination and enumeration of CTCs more robust. This microscope is a fully integrated solution that is not restricted to the analysis of CTCs, since it can be used for other applications involving the analysis of cell antigen expression by fluorescence microscopy, and it could also be updated to a brightfield microscope by using the same concepts described here, but with white light. The integration of both fluorescence and brightfield microscopy open the doors to automated liquid and solid biopsies. Moreover, further solutions for profilometry, particle tracking, and analysis of hybridized cytogenetic cell preparations could be implemented in the same platform after the inclusion of minimal changes in the software, making the presented device a versatile tool for surface characterization and biophysics research.

## Supplementary information

Supplementary Information File

## Data Availability

The datasets generated and/or analysed during the current study are available from the corresponding authors on reasonable request.
