# Supplementary material for: High-Throughput Automated Microscopy of Circulating Tumor Cells
Source: Sci Rep. 2019 Sep 24;9:13766. doi: 10.1038/s41598-019-50241-w (PMC6760523; doi:10.1038/s41598-019-50241-w)
Supplement: Supplementary file 1 — Supplementary Information File [file 41598_2019_50241_MOESM1_ESM.pdf]

High-Throughput Automated Microscopy of Circulating Tumor Cells
Supplementary Information

Carlos Aguilar-Avelar[1], Brenda Soto-García[1], Diana Aráiz-Hernández[1], Juan F. Yee-de León[1], Miguel Esparza[1], Franco Chacón[1], Jesús Rolando Delgado-Balderas[2], Mario M. Alvarez[3,4], Grissel Trujillo-de Santiago[3,5], Lauro S. Gómez-Guerra[6], Liza P. Velarde-Calvillo[1], Alejandro Abarca-Blanco[1,*], and J.D. Wong-Campos[1,7,*]

[1]Delee Corp., Mountain View, CA, 94041, USA.
[2]Departamento de Bioquímica y Medicina Molecular, Facultad de Medicina, Universidad Autónoma de Nuevo León, Monterrey, 64460, Mexico
[3]Centro de Biotecnología-FEMSA. Escuela de Ingeniería y Ciencias, Tecnologico de Monterrey, Monterrey, 64849, Mexico.
[4]Departamento de Bioingeniería, Escuela de Ingeniería y Ciencias, Tecnologico de Monterrey, Monterrey, 64849, Mexico.
[5]Departamento de Mecatrónica e Ingeniería Eléctrica, Escuela de Ingeniería y Ciencias, Tecnologico de Monterrey, Monterrey, 64849, Mexico.
[6]Servicio de Urología, Hospital Universitario “Dr. José Eleuterio González”, Universidad Autónoma de Nuevo León, Monterrey, 64460, Mexico
[7]Department of Physics, Joint Quantum Institute and Joint Center for Quantum Information and Computer Science, University of Maryland, College Park, MD, 20742, USA.
[*]Correspondence and requests for materials should be addressed to A.A. (email:alejandro@delee.bio) or J.D.W. (email:jwongcam@umd.edu)

1 Supplementary Figures

1.1 Autofocus

Figure S1 shows the flowchart of the autofocus algorithm implemented for the automated microscopy of circulating tumor cells (CTCs). Our approach considers the maximum value of the pixel intensity as focus descriptor and the unimodality of our focus function is ensured by considering the sum of pixels with the maximum value. This autofocus routine is called inside the stitching algorithm, every time the stage has reached the XY position where the new snap will be captured.

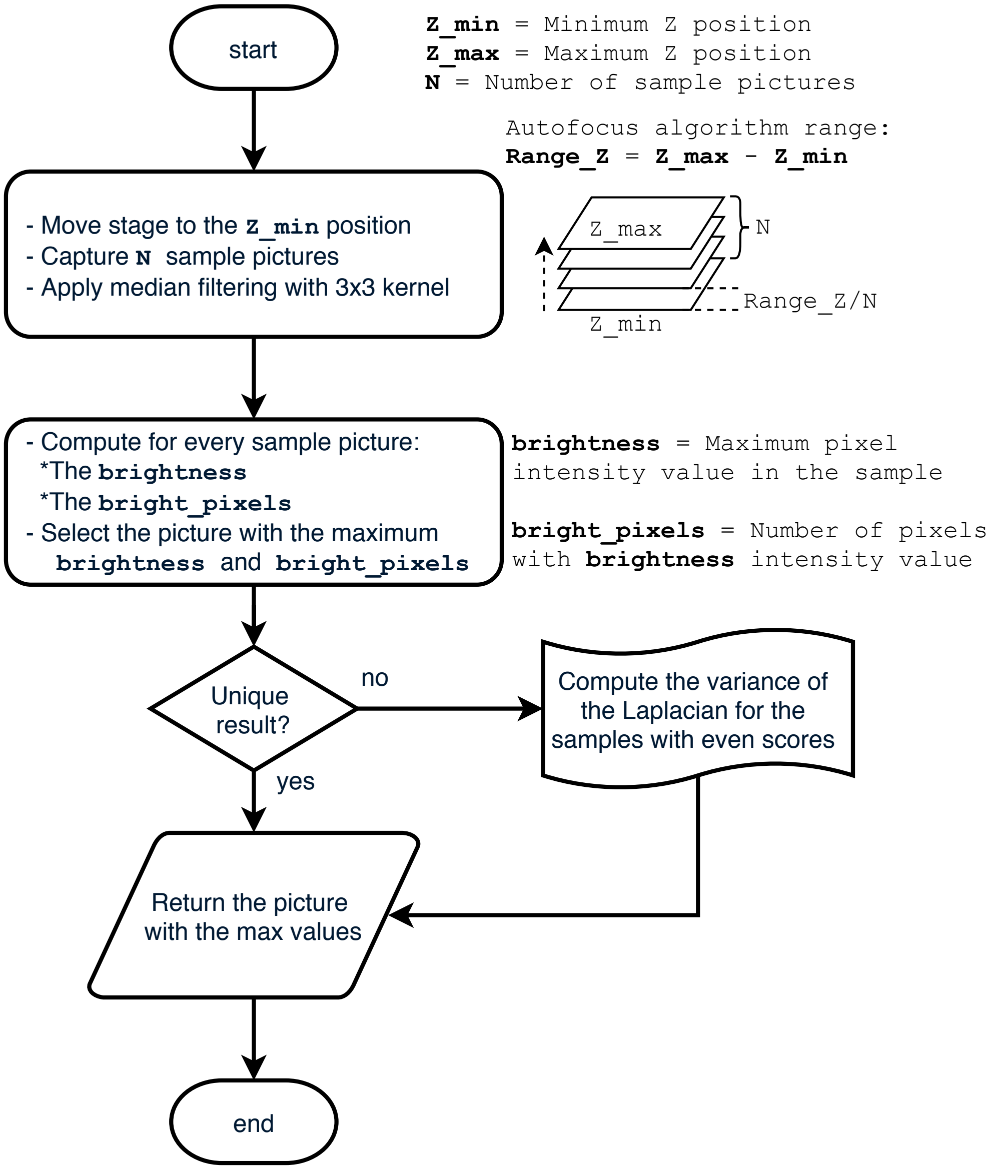

Figure S1. Flowchart of the autofocus algorithm. `N` sample pictures are taken equally distributed within the range `Range_Z`. A median filter is applied to samples to turn-off the hot pixels that may deteriorate the performance of the algorithm. The maximum brightness value `brightness` and the number of pixels with the maximum brightness value `bright_pixels` are computed for each sample picture. The picture with both the maximum `brightness` and number of `bright_pixels` is selected as the image of best focus.

In Fig. S2 we show the comparison of the maximum brightness and variance of the Laplacian values as focus scores for the autofocus algorithm. A Z-axis stack of 100 pictures (focus steps) with 1 μm of separation were taken from two different regions of a microscope slide with UV fluorescent polymer beads of about 6 μm of diameter. The brightness and variance of the Laplacian were computed for each sample picture of the two regions and the normalized resulting scores are shown in sub-figures (a) and (d) of Fig. S2, where the first region was selected to image just a few polymer beads and the second region to image a lot of them, respectively. Additionally, sub-figures (b) and (e) show the sample pictures with the best focus according to the variance of the Laplacian score, and sub-figures (c) and (f) show the sample pictures with the best focus according to the maximum brightness score. As can be seen in Fig. S2, the performance of the two scoring approaches was very similar for the region of the sample that was more populated with polymer beads, but in the case of the almost empty region, the performance of the variance of the Laplacian score is shown to be very poor, returning a maximum score for a picture that is out of focus; while the good performance of our brightness-based score remained.

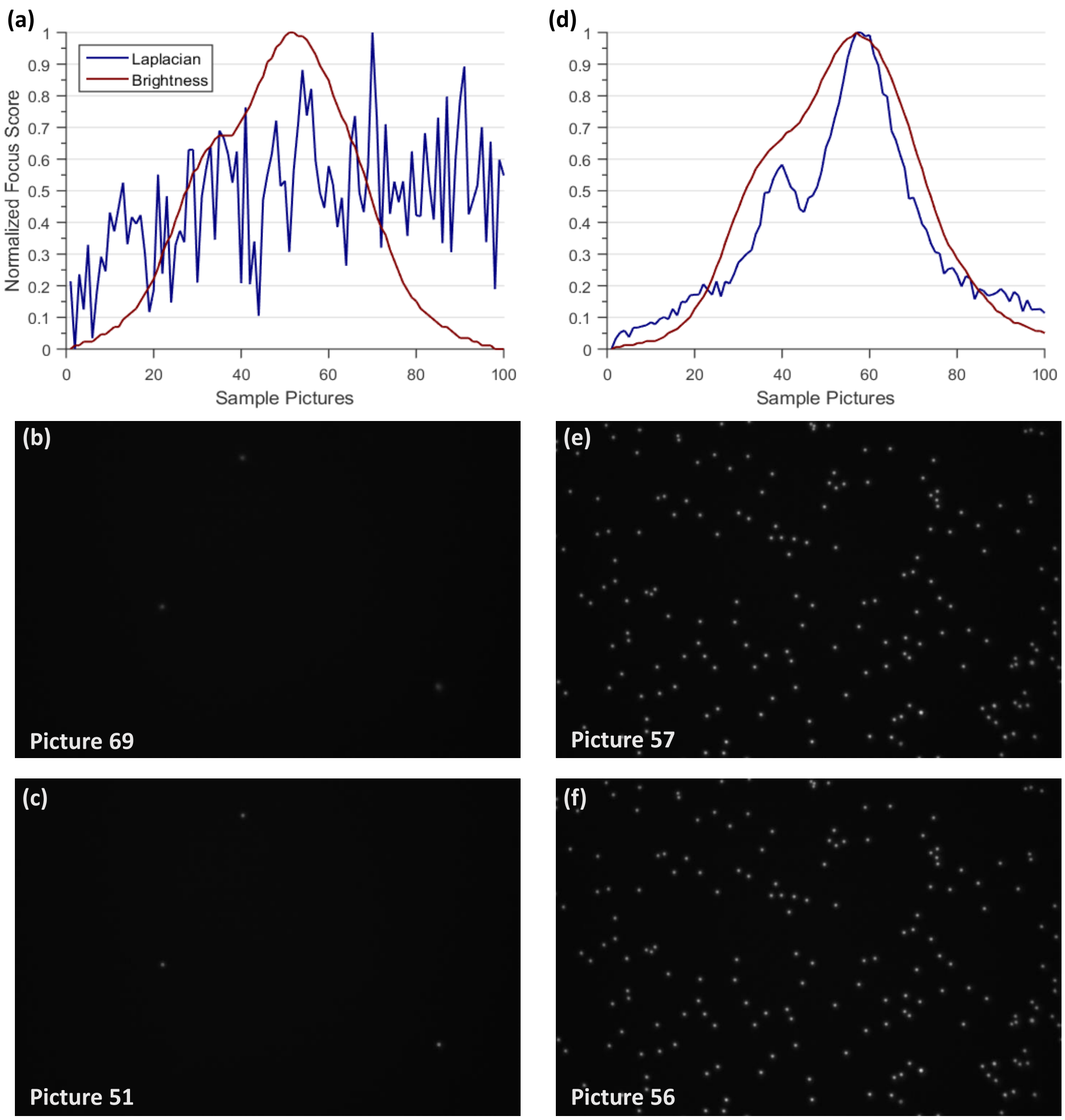

Figure S2. Comparison of the brightness and variance of the Laplacian values as focus function for the autofocus algorithm. Chart (a) shows the normalized focus scores computed for 100 pictures of a sample with a few UV fluorescent polymer beads. The snaps with maximum score were (b) for the variance of the Laplacian and (c) for the brightness-based score. Additionally, chart (d) shows the normalized focus scores computed for 100 pictures of a sample with a lot of UV fluorescent polymer beads. The snaps with maximum score were (e) for the variance of the Laplacian and (f) for the brightness-based score. All the sample pictures were captured with 10 ms of exposure time.

In Fig. S3 both the results of the stitching and autofocus algorithms are shown. Specifically, the results of a 7×1 mapping of a sample with and without autofocus are depicted. The sample contains UV fluorescent polymer beads of about 6 μm of diameter and it was deliberately tilted to exhibit the performance of our autofocus algorithm. The exposure time of each picture was 20 ms, 25 sample pictures were captured per snap within a range of 145.632 μm of the Z-axis, and the picture with maximum score returned by the algorithm described in Fig. S1 was selected for each snap. The entire mapping with autofocus was taken in less than one and a half minute.

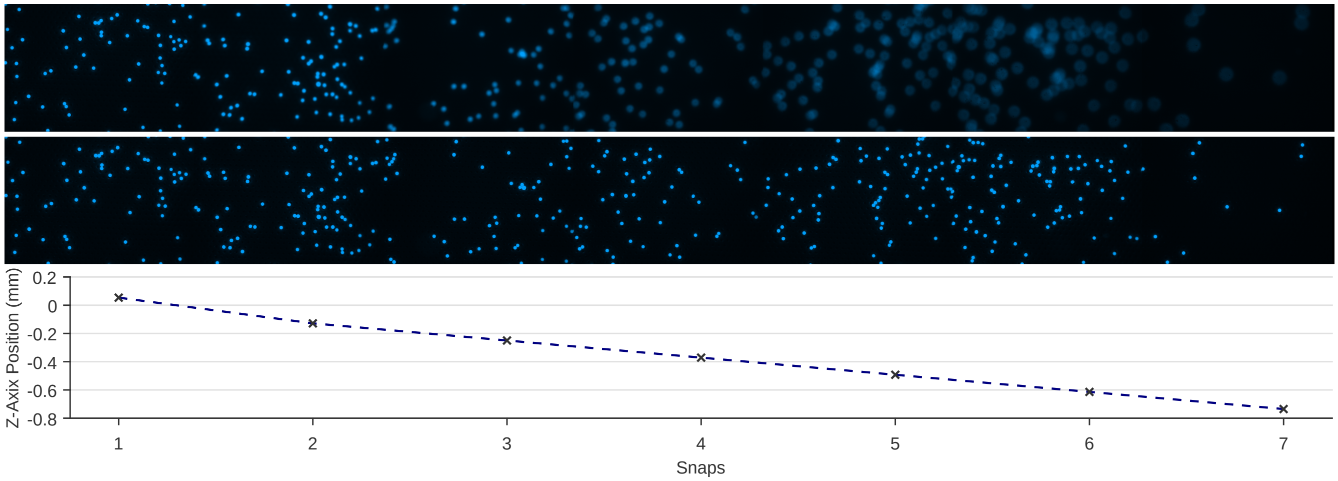

Figure S3. Stitching and autofocus algorithms performance evaluated on a tilted sample containing UV fluorescent polymer beads of about 6 μm of diameter. Top chart shows a 7×1 mapping captured without autofocus at zero position of the Z-axis. Middle chart shows the mapping of the same region of the sample obtained with the autofocus feature enabled. Bottom chart shows Z-axis relative positions obtained using the autofocus algorithm. All the sample pictures were captured with 20 ms of exposure time.

1.2 Excitation and emission bands

Figure S4 depicts the spectra of emission and excitation for the fluorophores we used, and the LEDs emission spectra. We notice that the green LED has a broad emission, which explains the drop of power at the output of the microscope objective.

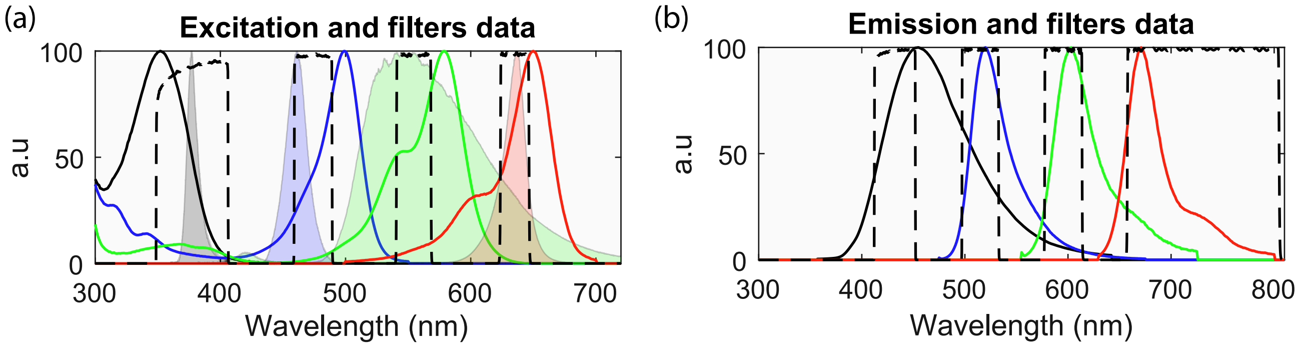

Figure S4. (a) Excitation bands for the four fluorophores. Solid lines depict the absorption wavelengths, solid colored plots the emission spectra of input the LEDs and the dashed lines show the wavelength dependent transmission bands. (b) Fluorophore emission spectra. The depicted plot shows the transmitted bands at four different wavelengths.

1.3 Bleed-through characterization

To quantify bleed-through of fluorescence emission from one channel to another, we measured the integrated fluorescence density in FIJI. We used four samples with cells that were stained with the four fluorophores (one fluorophore per sample) and each sample was imaged at all four channels. The exposure times were the same for the four samples, with values of 20 ms for the UV light, 50 ms for the blue light, 750 ms for the green light, and 50 ms for the red light. A single snap containing fluorescent events was captured from each sample and the software was used to apply a median filter with unitary radius, subtract the background, and measure fluorescence density. Fig. S5 shows the results bleed-through characterization of the four channels, where each column shows the labels that are related with the cell phenotypes expressed by each fluorophore and each row shows the light source that exited each fluorophore. The signal intensity of the channels that reflected crosstalk was expressed as a percentage of the intensity of the main channel.

Label / Light	Nucleus	CK	PSMA	CD45
UV	100%	0%	0.11%	0%
Blue	0.43%	100%	11.97%	0%
Green	0%	0%	100%	0%
Red	0%	0%	0.29%	100%

Figure S5. Results of the bleed-through characterization. Each column shows the labels that are related with the cell phenotypes expressed by each fluorophore and each row shows the light source that exited each fluorophore. The signal intensity of the channels that reflected crosstalk was expressed as a percentage of the intensity of the main channel. The exposure times were the same for the four samples, with values of 20 ms for the UV light, 50 ms for the blue light, 750 ms for the green light, and 50 ms for the red light.

1.4 Custom designed parts

In this section the schematics of the custom designed parts are found. In particular, the sketch of the 3D printed body of the microscope is depicted in Fig. S6 and the schematic circuit of the LEDs electronic driver can be seen in Fig. S7. The electronics is based on the pulse width modulation power driver CAT4101, which is controlled by an 8-bit microcontroller (Arduino nano). The power driver is able to provide up to 1A of current to each LED. Desired light intensity values are sent from a PC through serial communication and pulse-width modulated signals are generated by the microcontroller. This setup allows different brightness levels with a resolution of 8 bits.

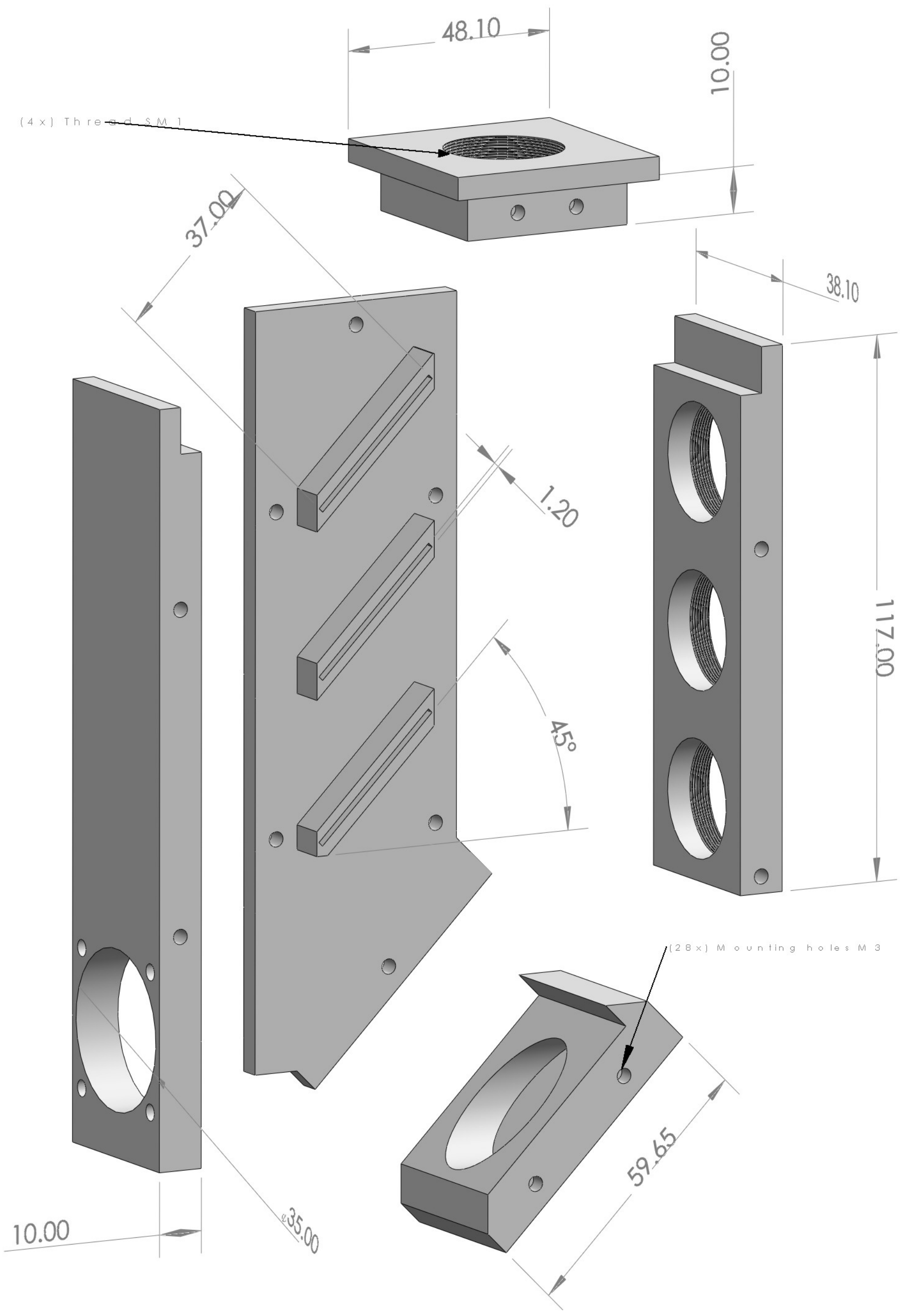

Figure S6. Draw of the light engine body.

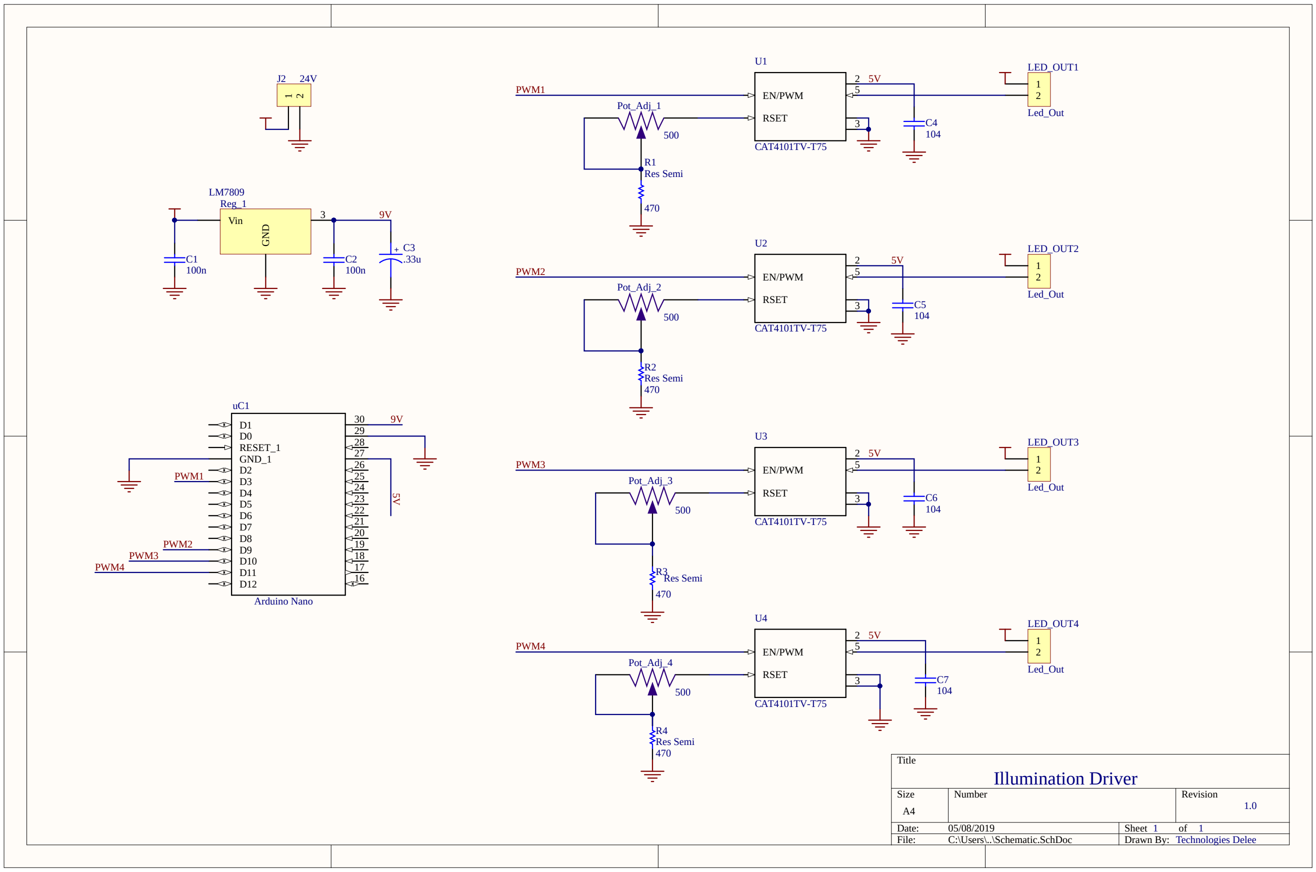

Figure S7. Schematic diagram of the illumination driver.
